# Agency as conversion process

**DOI:** 10.1007/s11186-022-09487-z

**Published:** 2022-08-01

**Authors:** Giacomo Bazzani

**Affiliations:** Department of Political and Social Sciences, University of Florence, via Pandette 32, Florence, Italy

**Keywords:** Agency, Capability approach, Conversion factors, Influence, Social change, Temporalities

## Abstract

Its importance for understanding social dynamics notwithstanding, the concept of agency is one of sociology’s more controversial ideas. The debate around this concept has mostly been developed at a theoretical level and the empirical studies tend to rely on socio-psychological interpretations of agency as a stable, inner force capable of influencing prospects, decisions, and behavior with little room for change in agency capacity. Social sciences, though, should take a more dynamic stance on agency and highlight the role of the different elements of the social context that can enable or hinder individual agency capacity. Prompted by recent developments of the Capability Approach, this article proposes a framework for the study of agency that defines individual agency as the result of a *conversion process* of personal resources shaped by *conversion factors*. Conversion factors operate at micro, meso, and macro levels of analysis, each of which can be oriented toward past experiences, present conditions, and future prospects. This article also seeks to analytically distinguish three types of agency outcome: *adaptation, autonomy*, and *influence*. Such a framework will facilitate the transformation of the slippery notion of agency into more tractable empirical phenomena which increase its analytical and critical capacity.

Agency is a “slippery” notion, but it is essential for understanding social dynamics. In general terms, it is used to refer to the forces that enhance, shape, or oppose the influence of social structure on social dynamics, though it remains one of the most controversial sociological concepts (see Alexander, 1993; Archer, 2000; Elder-Vass, 2010; Emirbayer & Mische, 1998; Sewell, 1992). In the case of human agency, the most widespread use of the concept of agency focuses on the human capacity to be the “perpetrator” of a given course of action (Giddens, 1984: 9). Without such a capacity, social dynamics would be totally predetermined by the social structure (Wrong, 1961), e.g., by institutional legacy and path dependency (Knöbl, 2010). But social structures are also often a resource for agency and may require an agency capacity to be maintained, e.g., racialized organizations (Ray, 2019). Overemphasizing human agency, however, risks creating an opposing naïve representation of societies based on individuals’ self-determination (Fischhoff et al., 1981)^1^.

The contrasting influences of structural context on agency have already been described in classical theoretical elaborations such as the theories of *habitus* (Bourdieu, 1977), structuration (Giddens, 1984), and morphogenesis (Archer, 1982; 1996; 2000); interpretations differ as to their mutual interdependence and the degree to which social structures are stable or fluid (Fuchs, 2001; Rutzou & Elder-Vass, 2019). However, this relationship cannot be reduced to a simple opposition between agency and structure or teleological and circular dynamics of co-determination between these two poles. Indeed, the co-determination of agency and structure implies neither a homogeneous distribution of the agency capacity nor the predictability of its effects on social structures. A proper understanding of agency should be able to account for this dynamic and often contingent nature without assuming a single and static source of agency. Given a set of projects and goals, for instance, the real agency capacity to achieve such goals can(not) emerge only under specific conditions. Indeed, the social context has a twofold influence on human agency: it can both hinder and enable agency capacity. The definition of the nature of elements of the social context that can both enable and constrain agency capacity remains a theoretical conundrum for agency research.

Considering the outcomes of agency from a normative perspective, agency is usually considered as a desirable capacity for human beings (Shogren et al., 2017), that is also worthy of support from public policy (Kosko, 2013; Niemiec & Ryan, 2009). However, the definition of agency outcomes poses similar challenges to those created by the nature of the elements involved. There are two main reasons for this: first, every definition of agency outcomes based on an already established list of specific capacities risks taking a normative stance over what the human being would or would not be (Kremakova, 2013; Zimmermann, 2006; 2018). Second, it is important to recognize that agency outcomes are going to be defined as an effect of agency capacity itself: if these outcomes were predetermined, the role of perpetrator of human agency would be downgraded and they could not be proper agency outcomes, but rather the outcomes of a different type of social process.

Given this challenging research scenario, empirical research on human agency has tended to focus on the socio-psychological characteristics that can account for the heterogeneity of individual success throughout life while neglecting its dynamic nature (Elder, 1994; Gecas, 2003; Hitlin & Kirkpatrick Johnson, 2015; Hitlin & Kwon, 2016). The principle of parsimony ensured that this type of research was oriented towards discovering a “golden factor” of agency based on a set of stable individual characteristics (e.g., self-efficacy, optimism). The main limit of this approach is that it assumes the stability of agency capacity as an individual characteristic with little room for individual change or for interventions for enabling agency capacity, other than education and socialization (Barnard et al., 2001; Cornwall, 2016). Moreover, this approach pays little attention to the social contexts in which agency decisions are taken or to the different – and contrasting – roles of enabler and constrainer that are embodied in the elements involved (Emirbayer & Mische, 1998; Shanahan & Macmillan, 2008).

To overcome these limitations of research on agency, this article proposes two contributions. First, it looks at the Capability Approach (CA) (Sen, 1985; 1993) and its recent sociological developments (Kremakova, 2013; Gangas, 2016; Hvinden & Halvorsen, 2018; Hobson, 2018; Zimmermann, 2018) and develops a framework for the study of agency based on the idea of *conversion process*. In recent years, the CA has aroused growing interest in many fields of research, such as human development (Alkire, 2005), health (Mitchell et al., 2017), social policy (Yerkes & Javornik, 2019), and child development (Gladstone, 2021), but its potential for agency research has not yet been developed. The conversion process is a social dynamic that converts personal and social resources into agency achievements which allow the various enabling or hindering roles of the elements involved in agency dynamics to be assessed. The elements that make up this process are the *conversion factors* that operate at the micro, meso, or macro level with different temporal orientations: the shadow of the past, the present, or the shadow of the future (Emirbayer & Mische, 1998; Bernardi et al., 2019). Conversion factors can act as *constrainers* of human agency – as per the traditional understanding of the role of social structures – or *enablers* of agency capacity.

Second, we define three types of agency outcomes that are informed by the nature of the relationship between the social context and agency dynamics. The first type of agency is *adaptation*: the personal capacity to be part of a social context and its structures that are considered valuable while allowing for different degrees of personal will. The second type of agency is *autonomy*: the capacity to personalize and maintain a distance from prescribed norms and social roles. The third type of agency is *influence*, the highest level of personal agency: the capacity to change social structures that constrain agency capacity and ordinary courses of action. These types of agency are shaped by the cognitive capacity of the “I” over the “Me,” which has the ability to construct different forms of the self (Mead, 2002 [1932]; Callero, 2003), as well as the processes of role making and role change (Goffman, 1968; Turner, 1990; 2001). However, the agency capacity of actively interacting with the social context is not limited to roles but also involves other social structures, such as institutions and norms. The three types of agency outcomes qualify the nature of their relationship with the social context and, in this way, define the effects of agency dynamics.

The discussion is organized into five sections. Section one explores the main theories and empirical research on agency and classifies them, according to the sources of agency capacity considered, as individual characteristics and social structures. Section two provides a more situated and dynamic account of individual agency based on the idea of conversion process, and offers some examples of how personal and structural resources can(not) be converted into agency achievements, e.g., positive illusions and virtual possibilities. The third section proposes a typology of the conversion factors that can be considered in agency research and provides some examples of its application to the case of hindering and enabling conversion factors that influence personal agency related to employment. Section four defines three types of agency outcomes on the basis of the different degrees of influence they have (not) on the social context: adaptation, autonomy, and influence. The concluding section discusses the contributions of the proposed framework for research on agency.

## The sources of agency

Despite the importance of the concept of agency for sociological research, its empirical investigation is less well developed than the theoretical debate that has flourished in recent decades. Some empirical phenomena exemplify the influential capacity of agency (e.g., social protests, entrepreneurship) or structure (e.g., social stratification), although the dynamic relationship between agency and structure is often overlooked. Moreover, the observation of social structure has typically been considered to be more “objective” and easy than the observation of agency capacity. For instance, the reproduction of social stratification through the inter-generational transmission of social positions is often cited as clear evidence of the presence of social structures that constrain individual agency (DiPrete, 2020; Stephens et al., 2007). Conversely, the presence of social mobility cannot readily be assumed to be a symptom of individual agency because it may also be the effect of macro-level social changes, within which individual agency has played a marginal role.

Individual agency is a complex social process whose outcomes cannot be easily deduced by observing the past because agency is “a temporally embedded process of social engagement, informed by the past (in its habitual aspect), but also oriented toward the future (as a capacity to imagine alternative possibilities) and toward the present (as a capacity to contextualize past habits and future projects within the contingencies of the moment)” (Emirbayer & Mische, 1998:963). The different temporalities embedded in agency can be analytically distinguished in three constitutive elements: iteration, projectivity, and practical evaluation. *Iteration* refers to the routine reactivation by actors of past patterns of thought and action; *projection* entails the human capacity to imagine possible future trajectories of action that cannot be deduced from the present; *practical evaluation* is the capacity to make practical and normative judgments among alternatives in the present (ibid.:917). The empirical research on agency usually makes a distinction between individual characteristics and structural conditions as the origin of agency. Both sources of agency are at the intersection of the three types of temporalities.

*Individual characteristics* have both “subjective” and “objective” dimensions (Hitlin & Long, 2009). The subjective dimension of agency has been measured as a sense of control (Ross & Mirowsky, 2013), mastery (Pearlin et al., 1981), self-efficacy (Bandura, 1997), self-esteem (Cast & Burke, 2002), optimism (Frye, 2012), expectations (Hitlin & Kirkpatrick Johnson, 2015), aspirations (Vaisey, 2010), and self-identification boundaries (Hitlin & Kwon, 2016). Evidence suggests that some of the subjective dimensions of agency may contribute to real individual achievements. Higher levels of achievements both at school and other educational attainments, for instance, occur among individuals with a higher sense of control (You et al., 2011) or mastery (Hitlin & Kirkpatrick Johnson, 2015). This type of agency is conceived as the psychological predispositions component of the iterational agency defined as a “set of internalized, partly pre-reflective beliefs” (ibid.:1435), that is also capable of sustaining humans’ capacity to project themselves into the future. The “objective” dimension of individual agency considers individual skills and resources (Clausen, 1991; Sewell, 1992). Individuals have different endowments of economic and social resources that can be mobilized for the achievement of personal goals or for facing life’s vicissitudes. Social capital, for instance, supports educational achievements (Behtoui & Neergaard, 2016) and careers (Lanford & Maruco, 2018; McArdle et al., 2007)^2^. However, neither the subjective nor the objective dimension of individual agency necessarily mean that a person can attain real achievements, because of structural and situational factors.

*Social structure* has a twofold influence on agency, be it subjective or objective individual agency, because it can silently reduce possibilities. Social structure influences individual objective agency with a different endowment of personal resources, but it also influences subjective agency through several channels already described in the literature. Social class, for instance, contributes to the shaping of expectations: youngsters and adults with a middle-class background show a greater ability for distinguishing themselves from others compared to their counterparts with a working-class background (Stephens et al., 2007), children born into less privileged families are less likely to aspire to university education (Reynolds & Johnson, 2011; Schoon, 2010) or to engage in long-term planning (Vaisey, 2010). Gender is also a source of subjective agency (Ross & Mirowsky, 2013), but school experiences (Bourdieu, 1984) and close friends (Schunk & Meece, 2006) also influence the personal sense of agency. Moreover, there is a second channel where the social structure influences agency through a “silent” production and reduction of possibilities. A more privileged social-class position, for instance, may offer greater opportunities for acting according to personal will. Conversely, individual agency often contributes to the reproduction of such structural conditions. In the case of expectations shaped by social class, for instance, individual agency may contribute to the reproduction of social stratification through the “socialization that constrains (or enables) people’s choices” (Hitlin & Elder, 2007:37).

## Conversion processes

Social stratification and other long-term social processes are part of the iterative side of agency, which often operates at the unconscious level. Projective and practical evaluative agency mostly take place in the form of deliberation when routines break down and people experience uncertainty over the future because what was expected as the outcome of the ordinary routine no longer seems to apply (Dewey, 1922 [1930])^3^. In such situations, imaginative capacity and “polyphonic micro-dialogues” (Burkitt, 2018:536) can play a central role in reconfiguring habitual elements, in rediscovering a “horizon of possibilities” that is to be found within each situation (Joas, 1996:133), and in orienting the course of action (Emirbayer & Mische, 1998; Mische, 2009; Ricoeur, 1991).

The reconfiguration process, however, still takes place within a course of action: decisions are not taken within an empty context, but are very much interdependent on the situation. Thus, while “emotions and personality traits—along with idiosyncratic personal histories, moral codes, and predispositions—influence the choices we make in emergent situations” (Hitlin & Elder, 2007:178), there is a whole more contingent situational context that influences the course of action and that cannot easily be reduced to either structural factors and long-term processes or a set of idiosyncratic personal characteristics. From a sociological standpoint, it would be extremely helpful to understand “what kinds of contexts provoke or facilitate them [actors] toward gaining imaginative distance from those responses and thereby reformulating past patterns through the projection of alternative future trajectories” (Emirbayer & Mische, 1998:1006). The process of reformulation implies no obvious connection between the availability of structural possibilities and personal resources and the individual’s agency capacity to consider and achieve such possibilities: this is due to situational factors which can play a prominent role in enabling or hindering agency capacity. For instance, an individual sense of agency and personal resources may not necessarily be converted into real agency capacity, even in a situation with structural possibilities. This possible disconnection can be observed in Sen’s distinction between “capability inputs,” which refer to the entire set of personal and structural resources, and two types of agency referred to as “freedom” and “achievement.” *Agency freedom* is defined as “one’s freedom to bring about the achievements one values and attempts to produce” (Sen, 1992:57), while *agency achievement* is “the realization of goals one has reason to pursue” (ibid.:56; see also Gangas, 2016).

This distinction is a useful analytical tool for observing the possible (dis)connections between personal and structural resources (“agency freedom”) and real individual achievements (“agency achievements”). Such (dis)connection may be the result of two types of misalignments between resources and situational constraints. On the one hand, personal resources related, for instance, to the individual sense of agency and the relative projections can be *positive illusions* (Taylor & Brown, 1994), because they may be based on unrealistic expected consequences of one’s own actions or characteristics, or those of others that are not converted into real achievements. This condition may still be due to personal resources such as cognitive bias, but it may also be related to a lack of structural or situational factors able to shape the “menu of options” (Burchardt, 2009). On the other hand, personal resources and social structure may also provide a range of *virtual possibilities* that are not converted into real achievements, because of more-or-less contingent situational factors. The contingent dynamics of the labor market and housing prices, for instance, are situational factors that are largely independent of both individual characteristics and social positions but they can definitely influence individual agency achievements by hindering or enabling personal plans. Empirical research has shown how the structural resources of family background are, in fact, more salient within the life-course agency when the labor market is poor (Heinz, 2009), but also the personal resource of planfulness capacity is more effective in a social context where viable options exist between school and work (Shanahan et al., 1997). These examples show that situational factors can determine whether or not individual resources and structural factors are converted into agency achievements through a *conversion process*^4^.

Agency achievements are the effect of the complex interplay that takes place between situational factors and long-term processes such as individual characteristics and structural factors explored within the literature on agency. The outcome of this interaction can never be predicted with complete accuracy and it can also give rise to some unexpected consequences: “very little is known from a sociological perspective about how and why people make decisions in the present about their lives … self-conceptions like self-efficacy undoubtedly matter, but their exact links to decision-making have not been established” (Shanahan & Macmillan, 2008:214). In order to disentangle this black box, research on agency needs to consider not just structural factors and psychological characteristics but also more situational elements at the decision-making level where structural and psychological forces interact with situational factors. Considering the individual ability for work-life balance, for instance, Hobson and colleagues (2014) note that while parents in different societies are protected by law against job loss or discrimination after returning from parental leave, there are marked differences as to how strictly these laws are enforced. These are due to both well-established cultural norms and other more situational factors (e.g., the recent public discussion in Sweden and the “use it or lose it” policy)^5^. Hobson suggests that “situated agency is a more sociological concept capturing the relational aspects of agency, the diversity in individual situations that shape agency freedoms, and the potential to convert resources into achievements” (2018:11). The concept of situated agency (Choi et al., 2019; Peter, 2003; Zimmermann, 2006) helps to consider the relational dimension of agency (Abbott, 2020; Burkitt, 2018): agency cannot be limited to stable or inner characteristics because it is “always agency *toward* something, by means of which actors enter into a relationship with surrounding persons, places, meanings, and events” (Emirbayer & Mische, 1998:973).

## Conversion factors

**Fig. 1:**
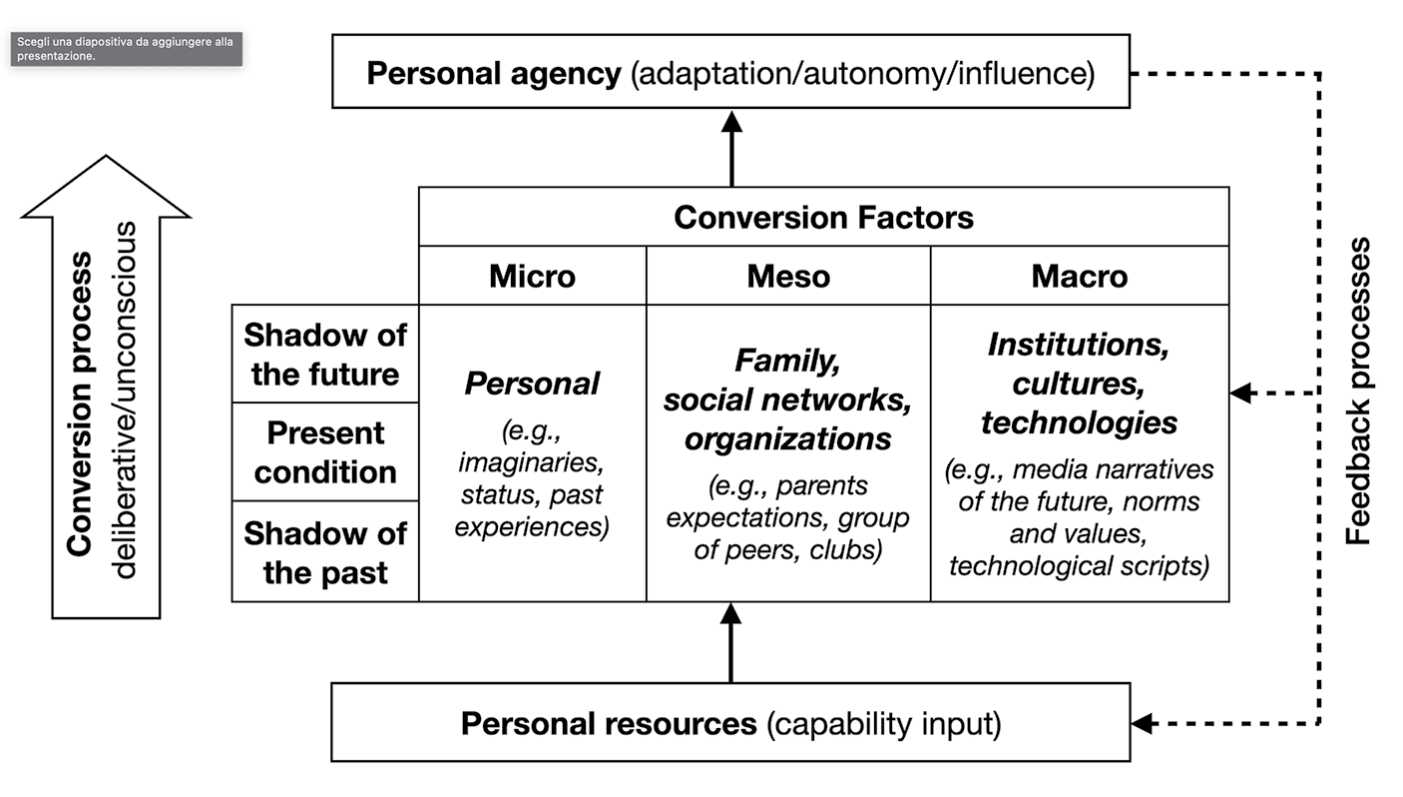
A schematic representation of the conversion processes with the different levels and elements involved (conversion factors), including their temporal orientation

When discussing the relational dimension of agency, the interaction between individual characteristics, social structure, and situational elements that may influence individual agency capacity needs to be considered. The capability input of personal and structural resources is not directly converted into agency achievements and agency freedom due to more-or-less contingent situational *conversion factors*. Conversion factors influence the extent to which a person can transform personal and structural resources into real agency achievements (Crocker & Robeyns, 2010). The set of capability inputs and conversion factors varies according to the social dynamic under observation. An exhaustive list of possible conversion factors that may be observed in a real-life situation is probably not possible, although various typologies have been attempted. Sen identified five main sources of conversion factors: personal heterogeneities, distribution within the family, differences in relational positioning, variations in social climate, and environmental diversities (1999; 2005; 2009); these were further elaborated by Robeyns into personal, social, and environmental conversion factors (2005; 2011). These sets of dimensions echo classical sociological distinctions though they are tainted by the normative assumptions regarding humans which are associated with the CA (Kremakova, 2013; Zimmermann, 2006; 2018)^6^. Recent elaborations of Sen’s framework have provided a more sociologically palatable approach to social dynamics (Kremakova, 2013; Gangas, 2016; Hobson, 2018; Hvinden & Halvorsen, 2018; Zimmermann, 2018). Apart from recent advances that consider the link between the CA and future temporality of action (Hobson & Zimmermann, 2022), such studies have generally given scant consideration to the different temporalities (not only future but also present and past) embedded in the course of action. Figure 1 proposes a schematic representation of the conversion processes with the different levels and elements involved (conversion factors), including their temporal orientation.

The iterative dimension of agency is embedded in elements belonging to the *shadow of the past* and the *present condition*, even though they often deploy their effects on future expectations (Reynolds & Johnson, 2011; Schoon, 2010). The *shadow of the future* embodies the projective agency (Beckert, 2016; Emirbayer & Mische 1998; Hobson & Zimmermann, 2022; Mische, 2009; 2014; Tavory & Eliasoph, 2013), while the practical evaluative agency takes place in the present condition with micro-dialogues that often involve elements which also belong to the past and future temporalities (Burkitt, 2018; Tuckett, 2017; 2018). The conversion processes mediate the relationship between resources (capability input) and agency (achievement and freedom) and they contain several elements that may be involved as conversion factors.

The conversion factors are presented as a typology organized according to their level (micro, meso, or macro) and their temporal orientation (shadow of the past, present, shadow of the future)^7^. The typology of the conversion factors cannot include every element that occurs in conversion processes in every specific situation. The schema provides a meta-theory of agency and may assist researchers by encouraging them to consider the elements involved and their interrelationship, and to elicit specific research questions on agency dynamics. Some elements of the schema, such as past experiences and institutions, can be considered either as resources or as conversion factors, depending on the specific research question. Resources and conversion factors are not distinguished by their nature but by the role they play in the specific agency dynamic (Zimmermann, 2006; Salverda et al., 2009; Hobson, 2014). It is “a question of the researcher’s perspective”: the researcher must judge which type of relevant variance (conversion factor) should be considered from among the available personal and structural “sources of capability input” (Hvinden & Halvorsen, 2018:874). We suggest that conversion factors can be thought of, in general terms, as the situational variance mediating the likelihood that the personal and structural resources available within a given target group become real agency achievements or freedom.

Conversion factors may not have any specific influence on the process of conversion of personal resources into agency—they may have a *neutral* result—or they may influence the process by *hindering* or *enabling* the conversion. Indeed, depending on the specific conversion process considered, some of the conversion factors do not play a role in the dynamic; therefore, they are neutral. For instance, while a peer group can support many agency achievements, in the case of an individual finding a job, they cannot play a role and are therefore neutral to this particular conversion process. Whether specific elements play a role or remain neutral in each conversion process cannot be defined once for all cases, as such an evaluation is part of the specific research question. In the next paragraph, the three levels of analysis and their temporal orientations will be explored and some examples will be provided of hindering and enabling conversion factors that influence personal agency related to the employment condition.

Personal characteristics are *micro-level* conversion factors. The shadow of the past contains past experiences that may influence agency capacity. The case of long-term unemployment, for instance, shows how these cumulative experiences together with the present employment condition undermine prospects as well as the capacity of envisioning a different future for themselves, thereby reducing individual efforts in job seeking (Lindsay, 2010). Conversely, personal imaginaries and aspirations to career and success can be a powerful source of agency, even in adverse economic conditions or when personal resources are lacking (Salazar, 2014; Van Heelsum, 2017). Future projects and aspirations, however, are always developed in interaction with others in a relational context (Bandura, 1989; Burkitt, 2016) – that can be considered as *meso-level* within the framework – that is primarily linked with the close network of family members and relevant others. For instance, career expectations of the family of origin are a shadow of the future that often shapes individual plans (Lund, 2018); loyalty-focused past-and-present interaction patterns of their ethnic group may inhibit youths from moving away from their neighborhoods, even when they offer fewer employment opportunities than others (Szalia & Schiff, 2014). Organizations may also elicit individual aspiration, as in the case of employees’ aspiration in some French businesses (Zimmermann, 2011; Lambert et al., 2012). However, given the same available resources, the capacity to aspire as an agency achievement may also be shaped by *macro-level* cultural factors, such as utopia and norms (Appadurai, 2004; O’Brien, 2015; Swidler, 1986, 2001). For instance, cultural repertoires may enable or hinder the aspiration to class mobility (Silva & Corse, 2018), but media-dominated narratives of the future also often influence individual decision-making processes, underlining prospects of economic and employment concerns over family plans (Guetto et al., 2020; 2022; Vignoli et al., 2020; 2022). Institutions play a major role at this macro-level of influencing conversion factors: policies may be a crucial conversion factor to achieving real gender equality in work-life balance and to enhancing women’s career opportunities (Hobson, 2014, 2018).

The meta-theory provided can help research on agency to explore a situated account of agency capacity and avoid reification of some stable individual characteristics. The conversion factors assist in defining which elements enable or hinder agency capacity; however, they cannot predict whether or which type of feedback process the agency outcomes will have on the initial conditions (Fig. 1). Indeed, an additional challenge of agency research is the definition of agency outcomes and their recursive capacity. Given the recursive co-determination of agency and structures (Archer, 1982; 2000; Elder-Vass, 2010; Giddens, 1984; Sewel, 1992), every agency outcome can become a resource for further agency achievements. This does not imply any direct circularity between agency achievement and resources, because agency outcomes cannot influence the conditions of their initial emergence: the social context under which agency took place may remain the same even after personal agency achievements. In the next section, we will define three types of agency outcomes on the basis of the different degree of influence they can have (not) on the social context.

## The outcomes of agency

In their socio-psychological work, Hitlin and Elder (2007) proposed a classification of the different types of agency based on the types of action they embodied. This classification partly echoes the three types of temporal orientation adopted by Emirbayer & Mische (1998). Existential, pragmatic, identity, and life course are the different types of agency classified on the basis of their different analytical scope and temporal orientation (Hitlin & Elder, 2007). Sociological interest in agency, though, should be oriented towards disentangling the complex interplay between resources and agency: agency has feedback processes which impact both conversion factors and the resources available (Fig. 1). Agency is more a matter of degree and “variance” (Fuchs, 2001) than “an ‘on/off’ capacity or condition” (Reich, 2002:93).

We propose to analytically distinguish three types of agency outcomes on the basis of the different degree of influence they have on the social context: *adaptation*, *autonomy*, and *influence*. These three types of agency outcome were developed from Sen’s three types of achievements (“well-being”, “agency” and “democracy”) and from Hvinden and Halvorsen’s (2018) elaboration of the key sets of values for “being in society” for persons with disabilities (“security”, “autonomy” and “influence”). However, compared with previous elaborations, our objective was to define these types as neutrally as possible without the normative stance on human beings that is often associated with the CA (Kremakova, 2013; Nussbaum, 2000; Robeyns, 2005; Zimmermann, 2006). While there may be a general consensus that certain basic rights, such as health and safety, are pre-conditions for greater human development, the exact outcomes of agency capacity cannot be established conclusively for every individual or group in all situations (Hvinden & Halverson, 2018; Trani et al., 2011). From a sociological standpoint, the analysis of agency outcomes should avoid any normative definition of human beings and be primarily oriented towards describing how agency capacity relates to the social context involved.

Being part of social institutions and social groups often requires a high degree of individual agency. *Adaptation* to the social context involved, for instance, can be a hard task for newly arrived migrants who require a high degree of personal agency. They may intentionally engage much of their energies and time for a long period in learning the new language, understanding the functioning of the public services, and creating a social network which also includes natives (Choi et al., 2019). A similar problem may also be faced by highly skilled workers: for young scholars, for instance, being recognized as an expert by their peers can require a lot of personal effort and time. Structural obstacles for inclusion may well be part of a given social context but the same context also offers some key conversion factors; e.g., laws and rights, volunteer groups, social subsidies, and supportive social networks (Portes et al., 2009). Adaptation would also be a central process for identity construction for “insiders”: group membership and of the performance of the social roles are crucial activities for the construction of the self that can require sustained agency efforts (Elias & Scotson, 2008; Goffman, 1968). Adaptation agency shows that achieving social positions and performing social roles can also require agency efforts, especially for marginal groups (Jackson, 1998). In such a case, adaptation means achieving one’s own goals in an unfavorable context that is exactly the opposite of lowering one’s aspirations (Semmeror & Schallberger, 1996). Adaptation does not imply the ability to influence the social context, although it can require a high level of personal agency and sustained effort if it is to lead to the accomplishment of personal plans or desires.

*Autonomy* represents individuals’ capacity to distance themselves from the roles, norms, and institutions of their social context. While social norms and roles influence agency capacity, the self can escape from the logic of over-socialized individuals and deviate from what is expected from them (Roessler, 2015; Taylor, 2004). The autonomy agency capacity reflects the cognitive capacity of the “I” over the “Me”: the “I” is the active part of the self that, in dialogue with the differently socialized “Me”, can always construct different forms of the self (Mead, 2002 [1932]; Callero, 2003). While norms, for instance, prescribe some gender roles, people are often capable of recognizing such roles and, more or less consciously, generating a new actualization of them within the role-making process (Brickel, 2006; Goffman, 1977; Turner, 2001). Autonomy essentially means being involved in the social context with a more or less extended ability to personalize the different roles prescribed (Turner, 2001) and to experience an embodied sense of self-determination. Goffman (1968) identified this process among individuals engaged in “strategic manipulation” aimed at de-emphasizing stigmatized identities. The most extreme type of role distancing is denial of the role. Truniger (1991) found a pronounced tendency among people who were unemployed after graduating from teachers’ college to distance themselves from “the unemployed” by describing themselves as “not really unemployed.” This autonomy agency involving distancing from prescribed roles is influenced by different levels of conversion factors beyond personal characteristics. For instance, Hobson (2014) described the role of policies and public debate in achieving tangible gender equality in work–life balance and women’s career opportunities, despite well-established legal norms. This type of agency, however, does not necessarily mean that macro-level cultural repertories are abandoned or do not play a role. For instance, research on second-generation migrants and female movements in North Africa has shown how cultural traditions can be strategically mobilized as a form of agency (Levitt, 2009; Mahmood, 2006; see also Swidler, 1986) and, more generally, cultural repertoires of individualization are a powerful source of autonomy capacity (Meyer & Jepperson, 2000). In contrast with the adaptation type of agency, autonomy agency requires the capacity to distance oneself from the social context, though it does not imply the capacity to influence such context.^8^

*Influence* is the highest level of personal agency capacity: it means the capacity to shape – more or less consciously – the social context that is the ordinary source of constraints for agency. This type of agency outcome overlaps with the traditional political mobilization of political parties or social movements (Dugan & Reger, 2006) that aim to influence the established order and rules but also belongs, for instance, to the capacity of collective action to manage the commons (Ostrom, 1990; Cleaver, 2007). The well-known conversion factors of this type of agency are the political institutions designed to elicit or promote democratic participation in political decision-making, e.g., political parties, deliberative assemblies, or even direct forms of participatory democracy (Curato, 2021; Fung, 2003; Sintomer, 2018). However, a change in institutions does not necessarily follow “ordinary”, familiar pathways: political, social, or technological revolutions, for instance, can be elicited by a small group of promoters but they can bring about massive changes in well-established institutions (Battilana et al., 2009; DiMaggio, 1988), such as niche innovation in transition studies (Schot & Geels, 2008). Influence agency capacity affects not only institutional change, but also role change. While autonomy agency can condition the process of role making, agency influence affects role change.^9^ As with other types of agency, conversion factors can also enable or hinder the implementation of role change (Turner, 1990). For instance, as macro-level conversion factors, revolutionary changes in work and family roles have been attributed to the transition from an industrial to a postindustrial society (Hage & Powers, 1992). Economic means and political power are often important resources for achieving agency outcomes. These resources can contribute to the role-making process of autonomy agency, as in the case of career expectations (Lund, 2018), but also to the processes of influence agency. Individuals and groups with more resources are often able to change roles and shape institutions with a more favorable balance of benefits and costs than are the less powerful (Turner, 2001), thus contributing to the reproduction of social inequalities and power asymmetry (Hitlin & Elder, 2007).

The three proposed types of agency are ideal-types of real agency capacities. In social dynamics, the three types of agency can be incremental, favoring spillover effects, or substitution effects between more or less feasible types of agency and agency domains^10^.

## Conclusions

Agency is a crucial dimension of social dynamics. Without agency, individual life and social dynamics would be reduced to unrecognizable, deterministic patterns, where the passage through life would be totally determined by the *habitus* and past experiences; psychological predispositions (Kohn, 1989) and social structures would force societies to remain the same for centuries. The idea of agency embodies sources of change for individual, organizational, and institutional trajectories, and their mutual interconnections. The analysis of agency, however, is better developed at the theoretical level than at the empirical level. The difficulties encountered in “measuring” agency capacity are due to its power to recursively shape the sources of constraint, but also to the theoretical conundrum of defining the nature of social elements that can be both enablers and constrainers of agency.

The aim of this article has been to better frame the notion of agency in social dynamics in order to strengthen its analytical capacity. In particular, we have tried to avoid common limitations of agency research that reduce the empirical analysis of agency to some stable proprieties of the social structure and psychological characteristics, or the simple voluntarism of personal will. Indeed, agency often expresses an (in)capacity to convert resources into real achievements. The processes of converting resources into agency cannot be directly reduced to, or deduced from, structural factors related to social position or individual psychological characteristics. Neither individual sense of agency nor structural conditions ensure agency freedom and agency achievements. Agency is a process more than a stable set of properties and is often shaped by more or less situational conversion factors that may have a hindering or enabling influence. The idea of agency as a conversion process is in line with a more situated account of agency (Choi et al., 2019; Hobson, 2014; Peter, 2003; Zimmermann, 2006). According to the pragmatist tradition agency capacity is not simply contingent upon the situation: the situation often does not provide merely the means and conditions for predetermined ends, but it structures patterns of response that can become the ground for agency (Abbott, 2020; Joas, 1996).

This article proposes a framework for a dynamic account of agency which focuses on the elements of social context that can enable or hinder agency capacity. To increase the analytical capacity of the concept of agency, we have proposed a set of conversion factors that provide a meta-theory which can be used to elicit specific research questions about agency dynamics and to open the black box of the (dis)connection between the available resources and agency achievements. Our proposal avoids the normative assumptions often associated with CA (Kremakova, 2013; Zimmermann, 2006; 2018). The proposed set of conversion factors may be useful for operationalizing the concept of agency in different settings, as well as considering the different levels of analysis (micro, meso, and macro) and their temporal orientation (shadow of the past, present, and shadow of the future). To our knowledge, this study is the first research framework that considers the three levels of analysis together with their different temporal orientations.

A further contribution of the article is the analytical distinction between three types of agency outcomes: adaptation, autonomy, and influence. Our proposal defines agency outcomes on the basis of their relationship with the social context involved in the agency dynamics. This distinction avoids the two risks implicated in a substantive definition of agency outcomes: (1) taking a normative stance over what constitutes a human being and (2) not overshadowing the situational nature of agency dynamics (Peter, 2003). The role of “perpetrator” of social dynamics embodied in agency definition (Giddens, 1984) emerges in each of these three types of agency, though the outcomes of this role shift according to the capacity of (1) being part of the social context, (2) expressing a degree of autonomy from it, and (3) influencing the social structures.

We believe this work can help in transforming the black box of the slippery notion of agency into more tractable empirical phenomena. This will increase the analytical and critical capacity of the notion of agency that can be further explored in several fields of study. Due to space limitations, the discussion in this article has been confined to the agency capacity of individuals: further research will focus on similar analyses for the agency capacity of non-humans, social groups, organizations, and institutions.

## Footnotes

1 Due to space limitations, this article focuses exclusively on human agency, notwithstanding agency capacity is not an exclusive property of human beings. Organizations (Beckert, 1999), institutions (Battilana et al., 2009; DiMaggio, 1988; Emmenegger, 2021), social groups (Sullivan et al., 2012), and non-humans (Harbers, 2005; Mackenzie, 1984; Latour, 1992) also have agency capacity.

2 This body of work, especially with regard to the subjective dimensions of agency, considers individual characteristics mostly with a cognitive focus, even though agency dynamics do not always occur consciously. The iterative dimension of agency is largely based on unconscious dynamics (Merleau-Ponty, 2012 [1945]) linked to social structure (Bourdieu, 1984), cultural influence (Cross & Gore, 2012), and the relational context of interaction and interdependence (Burkitt, 2018). These two-fold levels of agency are in line with a recent discovery of dual-system psychology: while we can control our conscious system, we are also motivated by an unconscious set of beliefs and emotions that often influence cognitive dynamics (Haidt, 2001; Kahneman, 2011).

3 For Dewey, the ordinary course of action is an unreflective flow of activities in which “habits do all the perceiving, recalling, judging, conceiving and reasoning that is done” (Dewey, 1922 [1930]:177), because “‘pure’ activity is for consciousness pure emptiness” (Dewey, 1922 [1930]:191). The ordinary unconscious course of action can be interrupted by the emergence of conflict between “different habits, or by the release of impulses; in such cases, the actor is confronted with a new and surprising situation” (Beckert, 2016:54). In this situation, “the objects experienced in following out a course of action”, that were not noticed before, start to “attract, repel, satisfy, annoy, promote and retard” (Dewey, 1922 [1930]:192): they become a problem for the ordinary course of action (routine) and the actor starts to be aware of them.

4 “The activation and expression of competing attitudes, the cognitive structures to which they are linked, and the behaviors that follow from them are elicited by social contexts” (DiMaggio, 2014:233–234).

5 Hobson suggests considering this shift in terms of “how entitlements are converted into a sense of entitlement” (2018:5).

6 These assumptions are found in Sen’s early work (1985; 1993) and to a greater extent in Nussbaum’s (1992; 2000; 2006) version of the CA which elaborates a list of universal capabilities required for human life.

7 We recognize that distinguishing the micro, meso and macro levels of analysis is matter of debate with different positions that vary from rational choice models (Coleman, 1990) to ethnomethodological tradition (Cicourel, 1981). The distinction here, though, does not concern the ontological base but is more pragmatically oriented towards creating a typology of elements that will assist in eliciting specific research questions. Technologies are a broad areas of conversion factors that cannot easily be confined to one level: they often provide a link between personal experience and macro dynamics (Muniesa et al., 2007).

8 O’Brien describes the different types of religious agency in terms of communal self-actualization, reflexive self-actualization, reflexive traditionalism, agentive orthodoxy, voluntaristic commitment, and privatized theism (2015).

9 For an introduction to the process of role change, see Turner, 1990; 2001.

10 For instance, migrant political participation can be a spillover of agency adaptation (Martiniello, 2006), while deviant school behaviors may be a case of agency substitutions: deviant behaviors may emerge as an autonomy agency that follows school failures and agency adaptation (Farrington, 2005).
